# Low PSA radiographic disease progression on C11‐choline PET

**DOI:** 10.1002/bco2.308

**Published:** 2023-10-25

**Authors:** Ahmed M. Mahmoud, Mohamed E. Ahmed, A. Tuba Kendi, Matthew Thorpe, Geoffrey B. Johnson, Irbaz Bin Riaz, Jacob J. Orme, Eugene D. Kwon, Jack R. Andrews, Daniel S. Childs

**Affiliations:** ^1^ Department of Urology Mayo Clinic Rochester Minnesota USA; ^2^ Department of Radiology, Division of Nuclear Medicine Mayo Clinic Rochester Minnesota USA; ^3^ Department of Medical Oncology Mayo Clinic Scottsdale Arizona USA; ^4^ Department of Medical Oncology Mayo Clinic Rochester Minnesota USA; ^5^ Department of Urology Mayo Clinic Arizona Phoenix Arizona USA

**Keywords:** C11‐choline PET, low PSA, prostate cancer

## Abstract

**Background:**

For men with prostate cancer, radiographic progression may occur without a concordant rise in prostate‐specific antigen (PSA). Our study aimed to assess the prevalence of radiographic progression using C‐11 choline positron emission tomography (PET) imaging in patients achieving ultra‐low PSA values and to evaluate clinical outcomes in this patient population.

**Methods:**

In a single institution study, we reviewed the prospectively maintained Mayo Clinic C‐11 Choline PET metastatic prostate cancer registry to identify patients experiencing radiographic disease progression (rDP) on C‐11 choline PET scan while the PSA value was less than 0.5 ng/mL. Disease progression was confirmed by tissue biopsy or response to subsequent therapy. Clinicopathologic variables were abstracted by trained research personnel. Overall survival was estimated using the Kaplan–Meier method. Intergroup differences were assessed using the log‐rank test. A univariate and multivariate Cox regression model was performed to investigate variables associated with poor survival after rDP.

**Results:**

A total of 1323 patients within the registry experienced rDP between 2011 and 2021, including 220 (16.6%) men with rDP occurring at low PSA level. A median (interquartile range [IQR]) of 54.7 (19.7–106.9) months elapsed between the time of prostate cancer diagnosis and low PSA rDP, during which 173 patients (78%) developed castration‐resistant prostate cancer (CRPC). Sites of low PSA rDP included local recurrence (*n* = 17, 8%), lymph node (*n* = 90, 41%), bone (*n* = 94, 43%) and visceral metastases (*n* = 19, 9%). Biopsy at the time of rDP demonstrated small‐cell or neuroendocrine features in 21% of patients with available tissue. Over a median (IQR) follow‐up of 49.4 (21.3–95.1) months from the time of low PSA rDP, 46% (*n* = 102) of patients died. Factors associated with poorer survival outcomes include advanced age at rDP, CRPC status, bone and visceral metastasis (*p* value <0.05). Visceral metastases were associated with decreased overall survival (*p* = 0.009 by log‐rank) as compared with other sites of rDP.

**Conclusions:**

Men with prostate cancer commonly experience metastatic progression at very low or even undetectable PSA levels. Periodic imaging, even at low absolute PSA values, may result in more timely identification of disease progression.

## 1 INTRODUCTION

Physical examination, serial prostate‐specific antigen (PSA) testing and periodic imaging are used to monitor for prostate cancer (PCa) disease progression. Increasingly, it is recognized that radiographic disease progression (rDP) occurs in the absence of rising PSA. A post hoc analysis of ARCHES, a trial comparing enzalutamide versus placebo in the hormone‐sensitive prostate cancer (HSPC) setting, revealed that 67% of patients experience rDP with non‐rising PSA.^1^ Similar findings have also been reported among those with castration‐resistant prostate cancer (CRPC). For instance, in PREVAIL, nearly 25% of enzalutamide‐treated patients had rDP without a concordant rise in PSA.^2^ These findings emphasize the importance of periodic imaging, regardless of PSA trajectory, to capture early disease progression in men with PCa.

It remains an open question as to how the absolute value of PSA should influence imaging practices, particularly among patients who achieve very low PSA levels with treatment. In general, achieving a low or undetectable PSA level has been associated with favourable long‐term outcomes after proctectomy, radiation therapy and systemic therapy.^3^, ^4^, ^5^, ^6^, ^7^, ^8^, ^9^ Conversely, some tumours evolve to low PSA secretion and demonstrate a very aggressive biology.^10^, ^11^ Thus, a mixture of favourable and poor prognoses exists among patients with low PSA.

In the era of conventional imaging, others have used a PSA cut point of 5 ng/mL to designate the entity of ‘low PSA secretors’. This value was selected, in part, based on its ability to detect transcriptionally defined treatment‐emergent small‐cell neuroendocrine prostate cancer (t‐SCNC). This small but important dataset of 15 patients harboured more adverse molecular risks (including RB1 loss and a low androgen receptor transcriptional signature) and had poorer overall survival as compared with the higher PSA secretors.^12^

The designation of ‘low PSA secretor’ is worth revisiting in the era of positron emission tomography (PET) imaging for PCa. Newer radiotracers, including prostate‐specific membrane antigen (PSMA) and C11‐choline, can detect rDP at lower PSA values.^13^, ^14^, ^15^, ^16^, ^17^ Our institution has a prospectively maintained C‐11 choline PET registry. The objectives of this study are to (1) catalogue the frequency and (2) characterize the clinical outcomes for patients who have radiographic progression by C‐11 choline PET imaging at ultra‐low PSA values (less than 0.5 ng/mL).

## 2 MATERIALS AND METHODS

### 2.1 Patient cohort and radiographic assessment

Following approval from the Mayo Clinic Institutional Review Board, we reviewed the prospectively maintained Mayo Clinic C‐11 Choline PET metastatic PCa registry to identify patients with PCa who experienced rDP on C‐11 choline PET scan at low PSA level (less than 0.5 ng/mL). This study determined rDP using previously published methodology.^5^ Trained nuclear radiologists provided the initial image interpretations. The blood pool corrected SUVmax value for each lesion was compared on pre‐ and post‐treatment scans. The data were analysed semi‐quantitatively. Interval change, morphologic characteristics and other factors were considered in the determination of progression. Any new C‐11 choline avid lesions were documented. Conventional imaging (such as an MRI, CT scan and bone scan) was used for correlation when available.

Disease progression on C‐11 choline PET was confirmed by tissue biopsy when feasible, conventional imaging or subsequent PET imaging. Patients in the C‐11 registry were additionally followed with regular physical examination, laboratory studies and serial imaging (C‐11 choline PET ± other imaging modalities). In general, patients within the choline registry are followed by urologists at Mayo Clinic with PSA and C‐11 choline PET every 3–6 months. Serial imaging is used for biochemical progression and response assessment in those with metastatic disease.

### 2.2 PET/CT imaging

C‐11 choline PET/CT was performed in accordance with the institutional standard clinical protocol (Discovery LS, RX, 690 or 710, General Electric). Each scan was performed from the orbits to the thighs with low‐dose, non‐contrast, free‐breathing CT images for attenuation correction and anatomic localization, with imaging beginning approximately 5 min after radiotracer injection.

### 2.3 Statistical analysis

Clinicopathologic variables were abstracted by trained research team members. Descriptive statistics were used for reporting categorical and continuous variables, including number (percentage), mean (standard deviation) and median (interquartile range [IQR]). Survival was assessed in months from the time of rDP. Overall survival analysis was performed using the Kaplan–Meier method, and between group differences (by site of progression) were assessed by the log‐rank test. A univariate and multivariate Cox regression model was used to identify factors associated with poor survival following rDP at low PSA level. Results were presented using hazard ratios (HRs) and 95% confidence intervals (CIs). All tests were two sided, and a *p* value of <0.05 was considered statistically significant. Statistical analyses were done using SPSS v.28 (SPSS Inc., IBM Corp., Armonk, NY).

## 3 RESULTS

### 3.1 Frequency of rDP at low PSA

A total of 1323 patients within the Mayo Clinic C‐11 Choline PET registry experienced rDP between the years of 2011–2021. Within this dataset, 220 (16.6%) men experienced rDP at low PSA (value <0.5 ng/mL). Of those experiencing rDP at low PSA, 84 (38%) patients had an undetectable (value <0.01 ng/mL) PSA level.

### 3.2 Clinical characteristics at initial diagnosis

Baseline clinicopathologic features for men ultimately experiencing low PSA rDP are listed in Table 1. The majority (*n* = 178, 81%) of men had recurrent disease after primary treatment with radical prostatectomy or radiation therapy. All had tumours available for pathologic evaluation at the time of their initial diagnosis. Only six (3%) patients had small‐cell/neuroendocrine PCa (SC‐NEPC) evident on their earliest biopsy. At the time of initial diagnosis, the median (IQR) primary Gleason score was 9 (7–9) and the median (IQR) PSA was 8.2 ng/mL (4.8–20.7).

**TABLE 1:** Patient characteristics.

Feature	Value (*n* = 220)
Median (IQR) age in years at time of diagnosis of prostate cancer	61.4 (56.1–67.6)
Median (IQR) PSA at time of diagnosis of prostate cancer	8.2 (4.8–20.7)
Family history of prostate cancer	
Positive	57 (26%)
Negative	163 (74%)
Initial Gleason score	
5	2 (1%)
6	7 (3%)
7	65 (30%)
8	34 (15%)
9	101 (46%)
10	11 (5%)
Histology at time of diagnosis of prostate cancer	
Adenocarcinoma	214 (97%)
Neuroendocrine	6 (3%)
Primary treatment at time of diagnosis of prostate cancer	
RP	147 (67%)
RT	31 (14%)
De novo metastatic	42 (19%)
Treatments received prior to low PSA rDP: androgen deprivation therapy	
Yes	181 (82%)
No	39 (18%)
Androgen receptor pathway inhibitor	
Yes	40 (18%)
No	180 (82%)
Chemotherapy	
Yes	70 (32%)
No	150 (68%)

Abbreviations: IQR, interquartile range; PSA, prostate‐specific antigen; rDP, radiographic disease progression; RP, radical prostatectomy; RT, radiotherapy.

### 3.3 Disease assessment at the time of low PSA rDP

A median (IQR) of 54.7 (19.7–106.9) months elapsed between the time of PCa diagnosis and low PSA rDP, during which time more than 78% of patients (*n* = 173) developed CRPC. A total of 42% (*n* = 92) had distant metastases prior to the occurrence of low PSA rDP. Systemic therapies received in the past included androgen deprivation therapy for 181 patients (82%), androgen receptor pathway inhibitors for 40 (18%) patients and chemotherapy for 70 (32%) patients.

Patterns of disease progression are outlined in Table 2. Osseous (43%) and lymph node (41%) metastases were seen most frequently when low PSA rDP occurred. Only 19 patients (9%) experienced progression that involved a visceral organ.

**TABLE 2:** Characteristics at time of low PSA rDP.

Feature	Disease status at the time of low PSA rDP	
	Hormone‐sensitive PCa (*N* = 47)	Hormone‐resistant PCa (*N* = 173)
Metastatic status prior to low PSA rDP		
M0	40 (85%)	88 (51%)
M1	7 (15%)	85 (49%)
Median (IQR) age in years at time of diagnosis of rDP	66.8 (59.9–73.5)	68 (61.3–74.1)
Median (IQR) PSA at time of diagnosis of rDP	0.25 (0.15–0.39)	0.14 (0–0.29)
rDP location		
Local only	5 (11%)	12 (7%)
Lymph node only	26 (55%)	64 (37%)
Bone ± lymph node	11 (23%)	83 (48%)
Visceral ± other	5 (11%)	14 (8%)
Histology at time of rDP		
Adenocarcinoma	8 (17%)	25 (14%)
Small cell/neuroendocrine	2 (4.3%)	7 (4%)
Not available	37 (78.7%)	141 (82%)
Median (IQR) follow‐up time in months from rDP diagnosis	82.3 (14.5–104.3)	48 (22.2–94.4)
Death		
Yes	7 (15%)	95 (55%)
No	40 (85%)	78 (45%)

Abbreviations: IQR, interquartile range; PCa, prostate cancer; PSA, prostate‐specific antigen; rDP, radiographic disease progression.

A new biopsy was obtained in 55 (25%) of the patients at the time of low PSA rDP, of whom 42 (76%) had evaluable results. Out of those biopsies, 33 (79%) demonstrated adenocarcinoma and 9 (21%) demonstrated small‐cell/neuroendocrine features.

### 3.4 Analysis of survival outcomes following low PSA rDP

**FIGURE 1:**
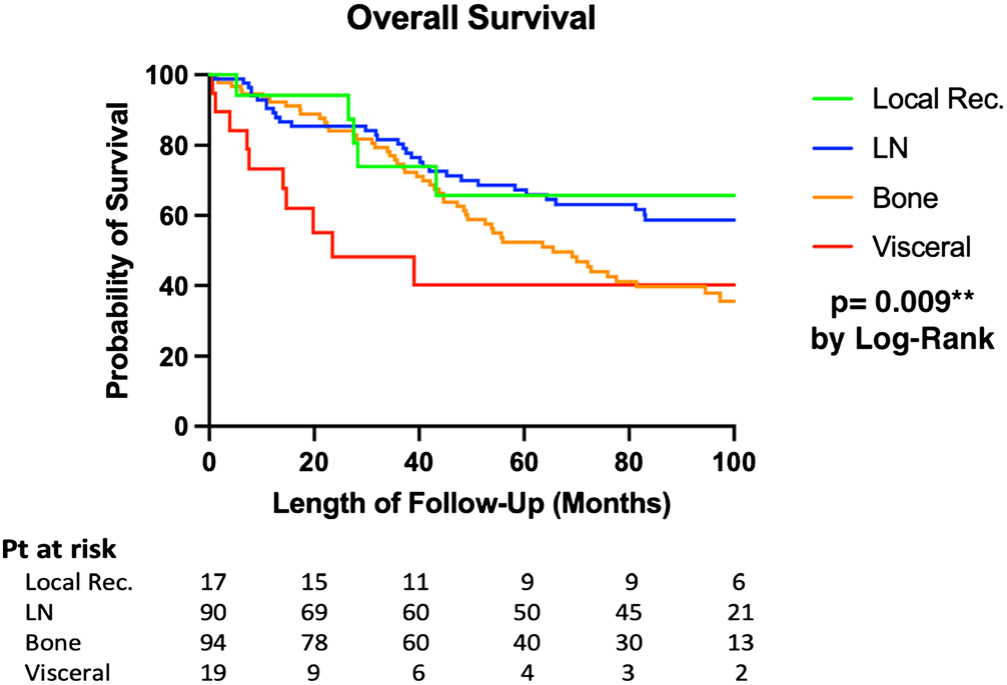
Kaplan–Meier survival curve (overall survival) by the sites of rDP.

Over a median (IQR) follow‐up of 49.4 (21.3–95.1) months from the diagnosis of low PSA rDP, 46% of patients (*n* = 102) died. Table 3 shows factors associated with poorer overall survival on univariate and multivariate analysis, including age at rDP (*p* = 0.014), CRPC disease status (*p* = 0.004), bone (*p* = 0.020) and visceral metastases (*p* = 0.013). Compared with other sites of rDP, visceral metastases were associated with poorer overall survival (*p* = 0.009 by log‐rank; Figure 1).

**TABLE 3:** Univariate and multivariate analysis of factors associated with overall survival.

	Univariate		Multivariate	
	Hazard ratio (95% CI)	*p* value	Hazard ratio (95% CI)	*p* value
Age	1.02 (1.01–1.05)	**0** .033*	1.03 (1.01–1.06)	0.014*
Gleason score	1.19 (0.98–1.45)	0.073	1.13 (0.92–1.37)	0.239
Prior treatment				
ADT	2.12 (1.13–3.97)	0.019*	1.07 (0.54–2.12)	0.851
ARPI	2.29 (1.47–3.55)	<0.001*	1.51 (0.88–2.59)	0.137
Chemo	1.63 (1.09–2.43)	0.017*	1.09 (0.65–1.82)	0.751
CRPC status	4.36 (2.02–9.40)	<0.001*	3.39 (1.47–7.83)	0.004*
rDP location				
Local	0.91 (0.57–1.46)	0.694	1.13 (0.69–1.84)	0.624
LN	0.88 (0.59–1.29)	0.503	1.39 (0.88–2.22)	0.155
Bone	1.75 (1.18–2.59)	0.005*	1.77 (1.09–2.86)	0.020*
Visceral	1.99 (1.01–3.97)	0.049*	2.61 (1.23–5.55)	0.013*

*Note*: Bold emphasis and asterisks denote statistically significant values.

Abbreviations: ADT, androgen deprivation therapy; ARPI, androgen receptor pathway inhibitor; CRPC, castration‐resistant prostate cancer; LN, lymph node; rDP, radiographic disease progression.

## 4 DISCUSSION

Prior studies have demonstrated the shortcoming of using PSA alone to assess treatment response in men with PCa. For those with metastatic disease, periodic imaging is needed to evaluate for evidence of disease progression. A deeper PSA response generally correlates with prolonged radiographic progression‐free and overall survival, which raises the question of whether frequent imaging is necessary for the patients who achieve a PSA nadir of 0.5 ng/mL or less.^7^, ^18^, ^19^ In our large, C‐11 choline PET registry, we found that nearly 1 in 6 progression events occur at low PSA level. In fact, many patients had an undetectable PSA level when rDP occurred. It is our view that routine imaging surveillance should not be omitted, even among patients achieving a very low PSA nadir, if the goal is to identify early disease progression.

This report builds upon post hoc analyses of clinical trial data from PREVAIL and ARCHES, which used conventional imaging for disease surveillance. At the time of PSA‐discordant rDP, these studies reported median absolute PSA values of 5.5 and 2.25 ng/mL, respectively.^1^, ^2^ In the current analysis, rDP was commonly detected at much lower PSA levels when more sensitive imaging modalities were used.

An important caveat should be acknowledged. Our cohort of patients received C‐11 choline PET as the primary imaging modality, which improves detection of PCa at low PSA values where conventional imaging may be less useful.^6^, ^8^, ^20^ Additional work is needed to understand whether earlier detection of rDP improves outcomes in metastatic PCa. There are multiple potential advantages. First, radiotherapy has emerged as a viable option for focal disease control in situations of oligometastatic progression.^21^, ^22^, ^23^ If imaging is delayed, the window of opportunity for metastasis‐directed therapy could be missed. Second, with earlier detection of more widespread disease progression, treatment modifications can be made before a patient experiences symptomatic or functional decline. Timely recognition of and reaction to progressive disease may delay or prevent worsening quality of life that commonly results from unabated cancer growth.

As might be expected, metastatic biopsies at the time of low PSA rDP were enriched for SC‐NEPC, despite these features being identified in only 2.1% of the earlier biopsies. The observed rate of SC‐NEPC in our study was 21%, which is similar to the 17% incidence reported by Aggarwal et al. in a prospective study that characterized treatment‐emergent disease variants in the era of modern androgen receptor signalling inhibitors.^24^ Histologic and molecular transition into a more aggressive entity is thought to be an important mechanism of therapeutic resistance.^25^ With time and selective treatment pressures, the cancer develops less reliance on androgen receptor‐dependent signalling. One hallmark of aggressive or ‘anaplastic’ PCa is low or absent PSA in the setting of metastatic progression, particularly in those with high volume or visceral metastases.^26^

Not all patients in our cohort had a poor prognosis. With over 4 years of follow‐up from the time of low PSA rDP, median overall survival still has not been reached. Reasons for the heterogeneity in outcomes are the inclusion of patients with HSPC and lead time bias. Additionally, most patients in this study had recurrent or metachronous PCa rather than de novo metastatic disease. As might be expected, the factors that were associated with poorer survival included age, CRPC disease status, bone and visceral metastases. When men with CRPC develop visceral metastases in the absence of PSA progression, the prognosis is best measured in months.^27^ Timely detection of visceral disease allows for biopsies and repeat molecular studies that can better inform subsequent therapies.

Our study is not without limitations. Disease progression was rarely confirmed with biopsy. Thus, the possibility of ‘false positive’ choline PET scan at low PSA cannot be excluded. Next, the need for an onsite cyclotron and the short half‐life of the C‐11 choline radioisotope limit its availability compared with other forms of metabolic imaging. Further studies should also evaluate the utility of PSMA‐based radiotracers in this setting which may be even more sensitive at low PSA levels. Third, molecular imaging is also associated with higher costs than conventional imaging; thus, cost–benefit analyses are needed. Finally, the impact of serial PET imaging on treatment outcomes in PCa should be prospectively evaluated in clinical trials.

In summary, our findings suggest that a PSA nadir of less than 0.5 ng/mL should not give treating providers a false sense of security. Routine imaging is still needed in this group of patients to detect early radiographic progression.

## AUTHOR CONTRIBUTIONS

*Conceptualization*: Ahmed M. Mahmoud and Mohamed E. Ahmed. *Writing—original draft preparation*: Ahmed M. Mahmoud and Daniel S. Childs. *Writing—review and editing*: Jack R. Andrews, Mohamed E. Ahmed, Jacob J. Orme, A. Tuba Kendi, Matthew Thorpe, Geoffrey B. Johnson, Irbaz Bin Riaz and Jack R. Andrews. *Statistical analysis*: Ahmed M. Mahmoud. *Supervision*: Eugene D. Kwon, Jack R. Andrews and Daniel S. Childs.

## CONFLICT OF INTEREST STATEMENT

This manuscript has no conflicts of interest to disclose.
